# Combating the triad of viral infections: tomato flu, monkeypox and COVID-19 by enhancing public health awareness

**DOI:** 10.1097/JS9.0000000000000174

**Published:** 2023-03-24

**Authors:** Vishnu Priya Veeraraghavan, Sreenidhi Prakash, Jyotsna Needamangalam Balaji, Lavina Prashar, Ullas Mony, Krishna Mohan Surapaneni

**Affiliations:** a Centre of Molecular Medicine and Diagnostics (COMManD), Saveetha Dental College and Hospitals, Saveetha Institute of Medical and Technical Sciences, Saveetha University; b Panimalar Medical College Hospital & Research Institute; c Departments of Biochemistry, Medical Education, Molecular Virology, Research, Clinical Skills & Simulation; d SMAART Population Health Informatics Intervention Center, Foundation of Healthcare Technologies Society, Panimalar Medical College Hospital & Research Institute, Varadharajapuram, Chennai, Tamil Nadu, India; e Department of Physiological Sciences, School of Medicine, Unza Ridgeway Campus, University of Zambia, Lusaka, Zambia

*Dear Editor*,

Amidst the world battling with the coronavirus disease 2019 (COVID-19) pandemic and an unprecedented surge in monkeypox infection, another newly emerging virus causing ‘tomato flu’ or ‘tomato fever’ in children below 5 years is being reported across India. It was first detected in Kerala and is now a cause of concern in other states of India as well. Although this rare viral infection is still in its endemic state and is considered nonfatal, appropriate containment measures should be taken to avert another devastating crisis^1^. Despite the fact that tomato flu is entirely a new infection, the similarity in the flu-like symptoms with COVID-19 and skin lesions as that of monkeypox infection creates a misconception among people^2^. Hence it is mandatory to create public health awareness about the triad: Tomato flu, COVID-19 and monkeypox to prevent misapprehension about the disease that may lead to diagnostic and treatment failure.

The most important aspect of public health awareness is information. The right information about tomato flu, population at risk, prevention, symptoms, diagnosis and follow-up should be delivered to the public. Although tomato flu presents with a few atypical manifestations, it is identified to be a possible variant of the long known hand–foot–mouth disease, which is caused by coxsackie virus A-16^3^. Owing to the fact that tomato flu primarily affects children below 5 years, the steps towards creating public health awareness should begin from schools, Anganwadi centres and local educational institutions that promote knowledge and awareness about the virus, mode of spread and preventive strategies. Parents and caretakers should be educated through door-to-door campaigns, small group gatherings, newspapers, booklets, awareness programmes and media about the symptoms of the virus, aetiology, mode of spread, Standard Operating Procedure, appropriate treatment measures and management protocols^4^.

About 64% of India’s population resides in rural areas. Lack of access to technological advancements, communication gap and unavailability of proper resources cause disease prevalence and mortality high among these people^5^. Thus, measures towards creating public health awareness should focus more on the rural population and devise disease outbreak and management plans as per the accessibility and usability of these people. Training the Primary Health Centre staff, Community Health Workers and Accredited Social Health Activists about the tomato flu, COVID-19 and monkeypox symptoms, detecting the skin lesions, differentiating the signs and symptoms from other infections, screening the vulnerable population, contact tracing, identifying the symptomatic patients and reporting the cases is inevitable to combat the spread of the virus as they serve as the main point of contact for any health issues in rural communities.

The government should focus on allocating funds and human resource for the utilisation of technologies like telehealth and mHealth facilities, which includes periodic phone calls to educate people about tomato flu, ensuring the health and sanitation status of the members of the family, SMS and reminders about tomato flu, COVID-19 and monkeypox prevalence and preventive measures and mobile phone apps that provide information about the disease and methods of early detection to enhance public health awareness and ensure healthy well-being of the people. Non-governmental organisations also play an important role in spreading health information, educating people about good health, disease prevention and spreading awareness about specific diseases, their prevalence and management strategies.

Since the world is still not out of the COVID-19 pandemic completely, subsequent disease outbreaks will lead to physical and mental stress among people putting the healthcare sector at stake. Regarding this, highly novel and efficient strategies to combat the possible outbreak of another disease are the need of the hour. Thus, we recommend a collective collaboration between governmental and non-governmental organisations together with healthcare providers and caregivers to empower the public about disease crisis and its management, which will create awareness among people and result in better health outcomes.

## Ethical approval

Not applicable.

## Sources of funding

No funding agency provided funding, and funding of any kind was obtained.

## Author contribution

V.P.V.: conceptualisation, data curation, and writing – original draft preparation; J.N.B., S.P., and U.M.: data curation and writing – original draft preparation; K.M.S.: conceptualisation, study design, data curation, writing – original draft preparation, reviewing and editing, and visualisation and supervision.

## Conflicts of interest disclosure

There were no conflicts of interest.

## Guarantor

Dr Surapaneni Krishna Mohan, corresponding author.

## Data availability

This correspondence is based exclusively on resources that are publicly available on the internet and duly cited in the ‘References’ section. No primary data were generated and reported in this manuscript. Therefore, data has not become available to any academic repository.
